# The Gut Microbiome and Substance Use Disorder

**DOI:** 10.3389/fnins.2021.725500

**Published:** 2021-08-31

**Authors:** Jordan T. Russell, Yanjiao Zhou, George M. Weinstock, Jason A. Bubier

**Affiliations:** ^1^School of Medicine, University of Connecticut Health Center, Farmington, CT, United States; ^2^The Jackson Laboratory for Genomic Medicine, Farmington, CT, United States; ^3^The Jackson Laboratory, Bar Harbor, ME, United States

**Keywords:** substance use disorder, addiction, microbiome, animal models, gut–brain axis

## Abstract

Substance use disorders (SUDs) remain a significant public health challenge, affecting tens of millions of individuals worldwide each year. Often comorbid with other psychiatric disorders, SUD can be poly-drug and involve several different substances including cocaine, opiates, nicotine, and alcohol. SUD has a strong genetic component. Much of SUD research has focused on the neurologic and genetic facets of consumption behavior. There is now interest in the role of the gut microbiome in the pathogenesis of SUD. In this review, we summarize current animal and clinical evidence that the gut microbiome is involved in SUD, then address the underlying mechanisms by which the gut microbiome interacts with SUD through metabolomic, immune, neurological, and epigenetic mechanisms. Lastly, we discuss methods using various inbred and outbred mice models to gain an integrative understanding of the microbiome and host genetic controls in SUD.

## Introduction

Substance use disorder (SUD) is a mental condition affecting the brain and is characterized in part by chronic dependence despite negative social, mental, and physical health consequences. Addiction represents the most severe form of SUD, with affected individuals often incapable of maintaining abstinence despite the will to discontinue substance use. Importantly, the effects of addiction can persist beyond cessation of substance use, suggesting that lasting physiological changes in the brain are involved (Koob et al., 2008). An expansive list of substance classes are related to SUD, ranging from legal forms, including alcohol, to illicit or controlled forms, including cocaine and opioids. Alcohol use disorder (AUD) remains the most prevalent SUD, however, poly-drug use within individuals is not uncommon (Lipari and Van Horn, 2017).

Addictive substances can affect multiple pathways controlling brain function. Alteration of the dopaminergic system is one common mechanism shared by all substances in the establishment of recurrent substance use (Nestler, 2005). Notably, substance misuse leads to increased levels of dopamine release and chronic signal imbalance in D2-like dopamine receptors in the ventral striatum, particularly in the nucleus accumbens (Koob and Le Moal, 2001; Nestler, 2005). Termed the mesolimbic pathway, substances of misuse operate to “hijack” the host reward system critical for pleasurable response and reinforcement to rewarding stimuli, as well as memory and emotional processes. Furthermore, the chronic use of these substances leads to neuroplasticity and structural reorganizations, and drug-specific changes in neurotransmitter transporters and receptors (Bliss et al., 2003; Kauer, 2004). Despite the central role of dopamine in SUD, other neurotransmitters such as serotonin and gamma-aminobutyric acid (GABA) also play an important role in SUD, depending on the dosage and frequency of substance use, as well as intrinsic biological factors such as host genetics (Prom-Wormley et al., 2017).

Previous human linkage studies have shown that children of individuals affected by SUD are at a greater risk for SUD, suggesting a genetic component to the risk of developing the disorder (Kreek et al., 2004). SUD involving cocaine confers the highest genetic risk of any substance, estimated at approximately 70% heritability (Goldman et al., 2005). Numerous genetic loci have been implicated in the heritability of risk for cocaine SUD, most notably genes involved in dopamine transport (*Drd2*, *Drd4*) and metabolism (*Dbh*) (Table 1). AUD also show strong genetic factors with variants in genes involved in alcohol metabolism (*Adh1b*, *Aldh2*) and diverse variants of smaller effect in dopaminergic systems (Table 1). However, discordant SUD rates found in identical twins suggest that genetics alone does not entirely explain the incidence of SUD (Agrawal and Lynskey, 2008). Aside from socio-economic factors studied extensively (Stone et al., 2012), there is intense interest in examining the role of the gut microbiome in SUD. The gut microbiota are a diverse population of microorganisms that constantly interact with the central nervous system (CNS) through complicated metabolomic, immune, neurological, and epigenetic pathways, making them potential sources of environmental influence on SUD.

**TABLE 1:** Substances involved in SUD and addiction, their mechanism of action and genetic risk loci for development of SUD/addiction.

Substance	Mechanism of action	Risk loci for developing SUD/addiction	References
Alcohol (ethanol)	Acts on multiple targets within the CNS including GABA synapse, glutamate signaling, and other neurotransmitters or receptors that indirectly control release of dopamine.	Variants in alcohol dehydrogenase (ADH1B); acetaldehyde dehydrogenase (ALDH2); small effect variants in reward pathway genes including dopamine receptor D2 (*Drd2*).	Boileau et al., 2003; Roberto and Varodayan, 2017; Edenberg and McClintick, 2018; Kranzler et al., 2019
Stimulants (e.g., cocaine, amphetamine)	Cocaine targets dopamine transporters, blocking dopamine re-uptake in dopaminergic neurons. Other stimulants such as amphetamines act to stimulate release of dopamine directly.	Dopamine transport and metabolism (*Drd2*, *Drd4*, *Dbh*); norepinephrine transporter (*Slc6a2*); 53 B-like family gene (*Fam53b*).	Noble et al., 1993; Volkow et al., 1999; Xu et al., 2000; Kahlig and Galli, 2003; Avelar et al., 2013
Opioids (e.g., heroin or pharmaceutical opioids)	Target the mu-opioid receptor MOR, leading to the activation of neurons containing MORs and subsequent dopamine release.	Variants in the mu-opioid receptor gene (*Oprm1*); potassium gated channels (*Kcnc1, Kcng2*); repulsive guidance molecule (*Rgma*).	Gelernter et al., 2014; Cheng et al., 2018; Valentino and Volkow, 2018; Zhou et al., 2020
Nicotine (tobacco)	Acts directly on nicotine receptors leading to dopamine release, or by activating other receptors indirectly.	Variants in the cholinergic nicotinic receptor gene locus (*Chrna1/b1*).	D’Souza and Markou, 2011; Hancock et al., 2018
Cannabis (marijuana)	Targets cannabinoid receptor CB1, indirectly stimulating dopamine release mediated through GABA and glutamate.	Variants near the forkhead box P2 gene (*Foxp2*); cholinergic nicotine receptor (*Chrna2*); epoxide hydrolase 2 (*Ephx2*).	Draycott et al., 2014; Demontis et al., 2019; Johnson et al., 2020

Although methods for investigating the microbiome in various diseases have made tremendous advancements in the last decade (Quigley and Gajula, 2020), studies of SUD in humans remain challenging because of the difficulty in controlling for complex experimental variables (for example, diet and host genotype). Model organisms such as mice provide excellent opportunities for studying the microbiome and its interaction with the host due to greater control over genetic and environmental confounders. In addition, mice of diverse but known genetic backgrounds can be harnessed to interrogate complex interactions between host genotype and the gut microbiota that may be important for risk of developing SUD (Poltorak et al., 2018). This review will provide a concise overview of recent insights on mechanisms of interaction between the gut microbiota and CNS, in addition to methodological considerations for studying the gut microbiome in SUD in animal models.

## The Gut–Brain Axis and Substance Use

The human gastrointestinal tract is home to a large community of microbiota on the order of trillions of cells. Likewise, the collective gene content of the microbiome encodes a staggering amount of functional potential, with an estimated 150 times greater number of genes than that of the human or mouse genome (Grice and Segre, 2012). Despite a core microbiome, fluctuations in composition are often observed after administering antibiotics, non-antibiotic medication, and diet intervention (Caporaso et al., 2011). This deviation from an individual-defined normal or “healthy” microbiome in the context of disease is termed dysbiosis, and a significant body of evidence has linked the dysbiotic microbiome as a contributor of disease and recently mental health (Shreiner et al., 2015; Capuco et al., 2020). There are multiple potential facets of gut host–microbe interactions to explain these disease associations, requiring much interdisciplinary research focus. Emerging evidence suggests prominent roles in host–microbe immune interaction, crosstalk between the gut and brain *via* neurotransmitters, and a role for bacterial metabolites including short-chain fatty acids (SCFA) in the pathophysiology of SUD. A summary of representative studies of the microbiome and SUD in animal models is provided in Table 2. For a comprehensive summary see the recent review by Angoa-Pérez and Kuhn (2021).

**TABLE 2:** Summary of representative studies using animal models in SUD and microbiome research.

Substance	Observations	References
Alcohol	Antibiotic treatment reduced voluntary alcohol intake by 70% in high-drinker rats; vagotomy led to similar reduction in alcohol consumption.	Ezquer et al., 2021
	Tigecycline antibiotic treatment reduced ethanol intake in male and female dependent/non-dependent C57B/6J mice.	Bergeson et al., 2016
	Transplantation of microbiota from alcohol-fed mice to controls led to similar alcohol withdrawal-induced anxiety behavior in recipients.	Xiao et al., 2018
Cocaine	Treatment with non-absorbable antibiotics for 2 weeks led to behavioral changes (enhanced reward, sensitization) in response to cocaine stimulation compared with controls in male C57BL/6J mice; microbiota depletion altered transcriptional activity in the nucleus accumbens; replacement of SCFAs reversed the antibiotic effect on behavior in response to cocaine.	Kiraly et al., 2016
Opioids	Intermittent and continuous treatment with morphine led to significant changes in the gut microbiota of male C57BL/6J mice; *Lactobacillus* were reduced and *Ruminococcus* were enriched after morphine exposure; antibiotic treatment led to increased drug tolerance and alterations in drug reward behaviors.	Lee et al., 2018
Nicotine	Nicotine altered the gut microbiota of C57BL/6 mice with sex-specific differences; nicotine led to decreased weight in males but not females; oxidative stress and DNA repair pathways were enriched in the microbiome after nicotine treatment; neurotransmitters (GABA) and precursor metabolites (glutamate) were significantly altered by nicotine treatment.	Chi et al., 2017
Cannabis	Differential gut microbiota composition in germ-free (Swiss Webster and B6.129P2(SJL)-Myd88^*tm1.1Defr*^) and conventional (C57BL/6 and B6.Cg-Lep^*ob*^/J) mice linked to expression of cannabinoid receptor CB1; modulation of CB1 expression is linked to gut permeability and leakage of pro-inflammatory LPS, also characteristic of the neuroimmune pathologies observed in SUD.	Muccioli et al., 2010

## Interactions Between the Microbiota and the Immune System in SUD

The gut microbiota produce an immense number of foreign antigens to which the host immune system must tolerate under normal homeostasis (Swiatczak and Cohen, 2015). The intestinal epithelium provides a physical barrier between the microbiota and immune cells, largely regulating the immune response to commensal microbes. Evidence suggests increased intestinal barrier permeability (leaky gut) in SUD, particularly AUD (Leclercq et al., 2014). Alcohol consumption has long been associated with developing a leaky gut through oxidative stress, which allows for increased translocation of bacterial products into the lamina propria. Here, microbial antigens can interact directly with gut-localized dendritic cells and macrophages and upregulate multiple pro-inflammatory cytokines production, leading to a heightened local and systemic inflammatory response.

Immune cytokines have modulatory effects on behavior. Levels of pro-inflammatory cytokines, including IL-1β, TNF-α, and IL-8, are increased in response to numerous substances of abuse (Meckel and Kiraly, 2019). Interestingly, this pro-inflammatory response to substance use is akin to the response to bacterial lipopolysaccharide (LPS) through signaling *via* toll-like receptor 4 (Northcutt et al., 2015). This pro-inflammatory response further implicates the interaction between the gut microbiota and the immune system in SUD pathology. It was observed that alcohol and opiates use leads to an enrichment of pro-inflammatory Proteobacteria in the gut. Likewise, anti-inflammatory responses are altered in instances of substance use which leads to an unbalanced neuroimmune response. Anti-inflammatory cytokines such as IL-10 have been shown to modulate and even reverse anxiety behaviors related to substance use (Patel et al., 2021). Furthermore, IL-10 could entirely diminish the escalation of alcohol intake by modulating GABA signaling in the amygdala. Thus, the link between the gut microbiota and the immune system *via* neuroimmune pathways are important research areas in SUD and addiction.

## Microbiota-Derived Neurotransmitters

Neurotransmitters intuitively play a critical role in the development and maintenance of all forms of SUD. Numerous bacteria have been described as capable of producing a number of neurotransmitters including GABA, serotonin, and dopamine (Yano et al., 2015; Strandwitz, 2018). A culture study showed that *Bacillus* species can produce dopamine. Furthermore, germ-free and antibiotic-treated mice have shown that the gut microbiota influences the production and turnover of dopamine outside the CNS (Strandwitz, 2018). Similar fluctuations in GABA levels are observed in mice treated with antibiotics (Yunes et al., 2020). *Lactobacillus*, *Bifidobacteria*, and *Bacteroides* species were identified to have the capability to produce GABA (Barrett et al., 2012; Strandwitz et al., 2019). Mice given specific GABA-producing *Lactobacillus* or *Bifidobacteria* have shown altered behavioral phenotypes (Yunes et al., 2020). Curiously, neurotransmitters produced by the gut microbiota may not under normal circumstances directly affect the brain, as whether these molecules cross the blood–brain barrier is still debated (Boonstra et al., 2015). However, increasing evidence has shown that the blood–brain barrier can fluctuate between states of increased permeability (Braniste et al., 2014). It may also be that the gut microbiota, rather than producing neurotransmitters themselves, affect neurotransmitter production or receptor expression indirectly *via* signals delivered through the vagus nerve (Bravo et al., 2011). Studies are ongoing to identify the full breadth of neurotransmitter-producing strains in the gut microbiome and the mechanisms by which microbial messages influence host behavior in the context of SUD.

## Gut Microbiome Mediated Epigenetic Mechanisms

Substance use leads to changes in gene expression in the brain in critical signaling pathways in the reward circuitry (Walker et al., 2018). Regulation of gene activation and repression is controlled in part epigenetically by adding and removing histone post-translational modifications. Common modifications include acetylation and methylation, which work to activate or silence gene expression, respectively. Importantly, these modifications are reversible; they can be added or removed by dedicated enzymes for this process, based on cellular cues. Histone acetylation, which leads to gene activation by opening the chromatin structure, increases at the nucleus accumbens in response to drug use for most substances (Renthal and Nestler, 2009). Alcohol is a unique case because the byproduct of ethanol metabolism is acetate itself. Acetate derived from alcohol metabolism post-consumption is readily incorporated into the brain, and in mice is associated with spatial memory and preference for the rewarding stimulus of alcohol (Mews et al., 2019). Inhibition or deletion of genes involved in acetylation, histone acetyltransferase (HAT), and histone deacetylase (HDAC) leads to alterations in sensitivity and drug-related behaviors (Cadet, 2016). These lines of evidence implicate the epigenetics of the brain in chronic SUD and addiction, and current research is focusing on the specific genetic loci affected by epigenetic alteration in response to substance use.

The gut microbiota produces large quantities of SCFAs, predominantly acetate, propionate, and butyrate, through carbohydrate metabolism and the breakdown of dietary fiber (Dalile et al., 2019). Butyrate, a potent HDAC inhibitor, can modulate host epigenetics through similar pathways as those affected by substance use (Simon-O’Brien et al., 2015). The microbial contribution to epigenetic modification may be important not only for current SUD but also the risk of developing SUD before initial drug intake, by predisposition toward the activation of addiction and reward pathways in the brain (Meckel and Kiraly, 2019).

## Studying the Microbiome in SUD Using Inbred Mice

The use of laboratory rodents to study the neurobiological aspects of SUD has been well documented, and the use of animal research to study the environmental effects of the microbiome is expanding rapidly (Fowler and Kenny, 2012; Meckel and Kiraly, 2019). Mouse models prove useful for investigating the interactions between host genetics and the gut microbiome in substance use. For example, C57BL/6 (B6) mice consume sufficient alcohol levels to mimic binge-like drinking episodes, making this strain a popular choice for modeling binge alcohol intake (Thiele et al., 2014). Further work in this model strain investigates links between alcohol consumption, the gut microbiota, host gene expression, and drinking-related phenotypes. Furthermore, rodent models of selectively inbred “high drinkers” have been proposed for the use of screening drugs aimed at reducing binge-like drinking behaviors (Crabbe et al., 2017). Using single or selectively inbred strains effectively isolates environmental variables of interest for experimentation by controlling for genetic variation in the host. Considering the complex diversity of the microbiota, isolating the microbial effects while controlling for host genetics is prudent. Despite the utility of inbred strains in isolating environmental effects, this method does not fully consider both the genetic and environmental complexity of SUD in humans, underpinning the critical issue of translatability of rodent models.

## Diversity Outbred Mouse Models

Much effort has been placed on developing recombinant-inbred mouse strains to capture greater genetic diversity in strains that are more applicable to complex disorders such as SUD in humans. In 2004, a large multi-institutional initiative led to the creation of the collaborative cross (CC) strains by crossing among a set of eight mouse strains which represent >90% of the genetic diversity of *Mus musculus* and its subspecies (Threadgill et al., 2011). Because CC mice originate from founder strains with defined genomes, this panel provides a high degree of genetic diversity that can be accurately mapped with genotyping techniques (Srivastava et al., 2017). Expanding further on the CC mouse panel are the diversity outbred (DO) strains, which provide even greater genetic and phenotypic diversity by outbreeding, producing unique individual heterozygous mice (Churchill et al., 2012).

There are numerous advantages to utilizing DO mice for mapping phenotypic traits to host genotype. Because they originate from the same founder strains as the CC panel, their genomes are well defined and suited for fine-detail trait mapping (Svenson et al., 2012). Furthermore, DO mice traits can be recovered from inbred CC lines to test for allelic effects. Research into benzene toxicity in DO mice revealed that harnessing this genetic heterogeneity in the mouse better reflected the diverse reactions to benzene exposure seen in humans (French et al., 2015). Efforts using heterogeneous stock (HS) mice, with similar genetic backgrounds as DO mice, have correlated genes with sex-specific ethanol preference and chronic consumption phenotypes, and similar results have been corroborated in DO mice (Hitzemann et al., 2020). In the context of SUD, DO mice may be used to study the interaction between host genes, the environment (i.e., microbiome), and phenotypic differences between strains that reflect similar genetic diversity in humans.

## Reproducibility Through a Controlled Environment

Despite considerable effort, reproducibility in microbiome research using animal models remains challenging. Careful considerations must be made when studying the relationship between genetics, phenotype, and the microbiome in an animal. Microbiome differences in mice can be seen based on external factors such as vendor, environmental and facility conditions, and co-housing with other mice (Justice and Dhillon, 2016). When controlling for genetics by using inbred strains, mouse-to-mouse variation in microbiome composition is observed. A consistent methodology plays a crucial role in the reproducibility of microbiome research in animals, as circadian rhythm can also have confounding effects for sampling at different times of the day (Thaiss et al., 2014). In order to reduce confounding individual variation of the microbiota, strategies for the rotation of mice and mixing of bedding may be used so that all mice in the experiment are normalized to the same common microbiota. Furthermore, greater reproducibility can be achieved by defining a standard microbiota composition for a given model or experiment (Witjes et al., 2020). Thus, experimental replication is more likely to be achieved by knowing the target microbiota important for obtaining similar outcomes.

Though defining a normal microbiota for an experiment or model organism may lead to a greater likelihood of reproducibility, it raises the question of translatability since a standardized microbiome is not realistic in humans. Here the concept of increased reproducibility is at odds with the fact that human disorders such as SUD are not simply defined systems. Just as genetically diverse DO mice better reflect the natural heterogeneity seen in humans, so too is there value in assessing natural variation in the microbiome. The advent of “wildlings” by implanting lab-strain mouse embryos into wild mice provides a useful tool for studying a more naturally derived microbiome in an effort to enhance translatability (Rosshart et al., 2019). Ultimately, the choice to standardize and how will depend on the experimental question; whether there is a necessity to study the full breadth of genetic and microbial heterogeneity such as that in humans, to discover interactions between host and microbe, or to isolate as best as possible specific genetic and environmental factors for studying direct effects.

## Discussion

Addiction and substance use research has seen renewed enthusiasm with a focus on the innate gut microbiota. Numerous studies have proposed a connection between the gut microbiota and the CNS *via* the gut–brain axis, providing a mechanism by which these microbes influence the host. Often drug use is associated with dysbiosis of the gut microbiome, and these changes may be critical to the establishment and maintenance of addiction by altering signals between the gut and the brain. Thus, a probiotic intervention has garnered much interest as a novel form of therapy for SUD. Recent evidence shows promising effects of probiotics in treating other mental disorders, including anxiety and depression, both of which are often co-morbid with SUD (Abildgaard et al., 2017; Hadizadeh et al., 2019; Kim and Kim, 2019; Liu et al., 2020). Fecal microbial transplant is also an area of intense research. Studies in mice have shown that transplantation of fecal microbiota of individuals diagnosed with AUD leads to altered social and anxiety behaviors and increased preference toward alcohol (Zhao et al., 2020). Reciprocally, transplantation of microbiota from healthy donors reduced anxiety and depressive behaviors in mice exposed to alcohol (Xu et al., 2018). More research is needed to validate the effects of FMT for other addictive substances and whether dysbiosis perpetuates the chronic nature of addiction. Future work will focus on untangling the mechanisms of gut microbiota modulation on the host addiction behaviors to identify target pathways and functions for potential probiotic therapeutics, such as those focused on SCFA production.

Gut microbiome dysbiosis is evident after chronic and acute substance use. However, more research is needed into whether the gut microbiome may also serve as a source of environmental risk for the development of SUD and addiction or be used as a predictor of future SUD. Furthermore, interactions between host genetics and gut microbiota deserve greater attention. Recent evidence has suggested that host genetics can have strong effects on the composition and function of the gut microbiome (Goodrich et al., 2014; Abildgaard et al., 2017; Kim and Kim, 2019; Korach-Rechtman et al., 2019; Liu et al., 2020). Using animal models from diverse genetic backgrounds will pave the way toward understanding complex interactions between genetic traits and the microbiota, especially given that genetically diverse mice respond differently to probiotic interventions (McVey Neufeld et al., 2018). Lastly, researchers must consider the balance between the reproducibility of using inbred mouse strains to test specific hypotheses versus the translatability of genetically diverse mice, as both methods will be critical to not only bolstering our understanding of the microbiome in SUD and addiction but also its applicability to a diverse human population.

## Author Contributions

JR drafted the initial version of the manuscript. YZ, GW, and JB contributed to and edited the final manuscript. All authors approved the final manuscript.

## Conflict of Interest

The authors declare that the research was conducted in the absence of any commercial or financial relationships that could be construed as a potential conflict of interest.

## Publisher’s Note

All claims expressed in this article are solely those of the authors and do not necessarily represent those of their affiliated organizations, or those of the publisher, the editors and the reviewers. Any product that may be evaluated in this article, or claim that may be made by its manufacturer, is not guaranteed or endorsed by the publisher.
